# Heterotopic ossification in a 7‐year‐old female patient treated with individualized homeopathy: A case report

**DOI:** 10.1002/ccr3.2547

**Published:** 2019-11-19

**Authors:** Dionysios Tsintzas, Atul Jaggi, Latika Jaggi, Seema Mahesh, George Vithoulkas

**Affiliations:** ^1^ Orthopaedic Surgeon Rehabilitation Centre General Hospital of Aitoloakarnania Agrinio Greece; ^2^ H3 Centre of Classical Homeopathy Nashik India; ^3^ Centre for Classical Homeopathy Bangalore India; ^4^ University of the Aegean Mytilene Greece

**Keywords:** classical homeopathy, heterotopic ossification, individualized therapy

## Abstract

Classical homeopathy can be included among the treatment options for congenital heterotopic ossification.

## Graphical abstract

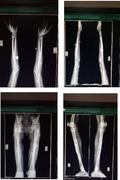

## 1 INTRODUCTION

We present a case report of a seven‐year‐old girl suffering from severe congenital heterotopic ossification. The patient received treatment with individualized remedies according to the rules of Classical Homeopathy, demonstrating steady improvement in both her clinical condition and the radiological findings over the next 2 years.

Heterotopic ossification (HO) consists of the formation of bone tissue at extraskeletal anatomical sites at the expense of local tissues, including muscle and connective tissue.^1^ The formation of lamellar bone in soft tissues, where bone normally does not exist, is also called myositis ossificans. However, it would be more accurate to describe the involvement of skeletal muscles as myositis ossificans and the involvement of soft tissues in general as ectopic or heterotopic ossification.^2^ These bony masses can lead to chronic pain, joint ankylosis, pressure ulcers, venous thrombosis, and many other health complications.^3^

Heterotopic ossification can be acquired, triggered by trauma, surgical procedures, spinal cord and brain injuries, extensive burns or long‐lasting immobilization.^1^, ^2^ Congenital HO is a very rare condition that occurs in pediatric patients and is regarded as an autosomal dominant disease with irregular penetrance leading to ectopic bone formation and motion disturbances.^4^ Morbidity and early mortality in children is due to respiratory complications and improper treatment of the lesions, with very few cases being cured by surgical intervention.^5^, ^6^

## 2 CASE HISTORY

**Figure 1:**
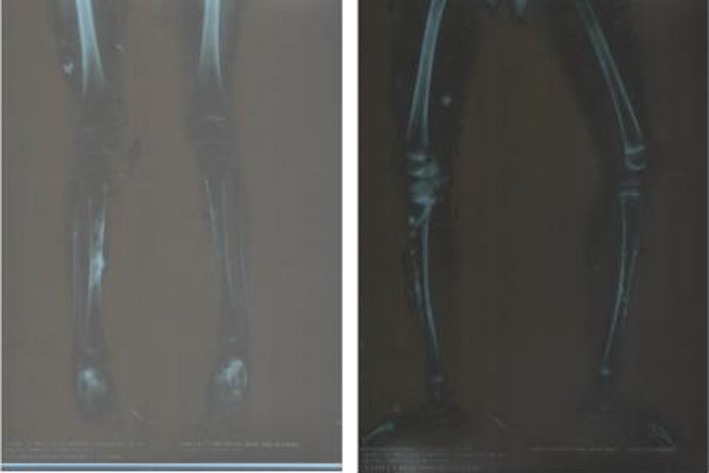
The first radiological findings, patient 2 y old

The patient of our case report is a 7.5‐year‐old girl who presented to the clinic with extensive clinical and radiological signs of heterotopic ossification. Five years ago, when the patient was 2.5 years old, the first radiological investigation showed extensive soft tissue calcification, with normal alignment and architecture of the bones and no evidence of fracture, lytic or sclerotic bone lesion (Figure 1). A skin biopsy from the lesions of both thighs showed the possible diagnosis of “calcinosis cutis.” At that time, the antinuclear antibodies (ANA) were positive and lactate dehydrogenase (LDH) was elevated (346 IU/L, normal range 81‐234 IU/L), indicating tissue damage. SGPT was 44.4 IU/L (normal 0‐31), SGOT was 43.5 IU/L (normal 0‐31), and ALP was 163 IU/L (normal 28‐78). Juvenile dermatomyositis was the diagnosis given after a dermatologic consultation, and the patient was prescribed topical corticosteroid ointments and oral methotrexate. There was no improvement in the clinical condition of the patient, so she discontinued the treatment 4 months later.

**Figure 2:**
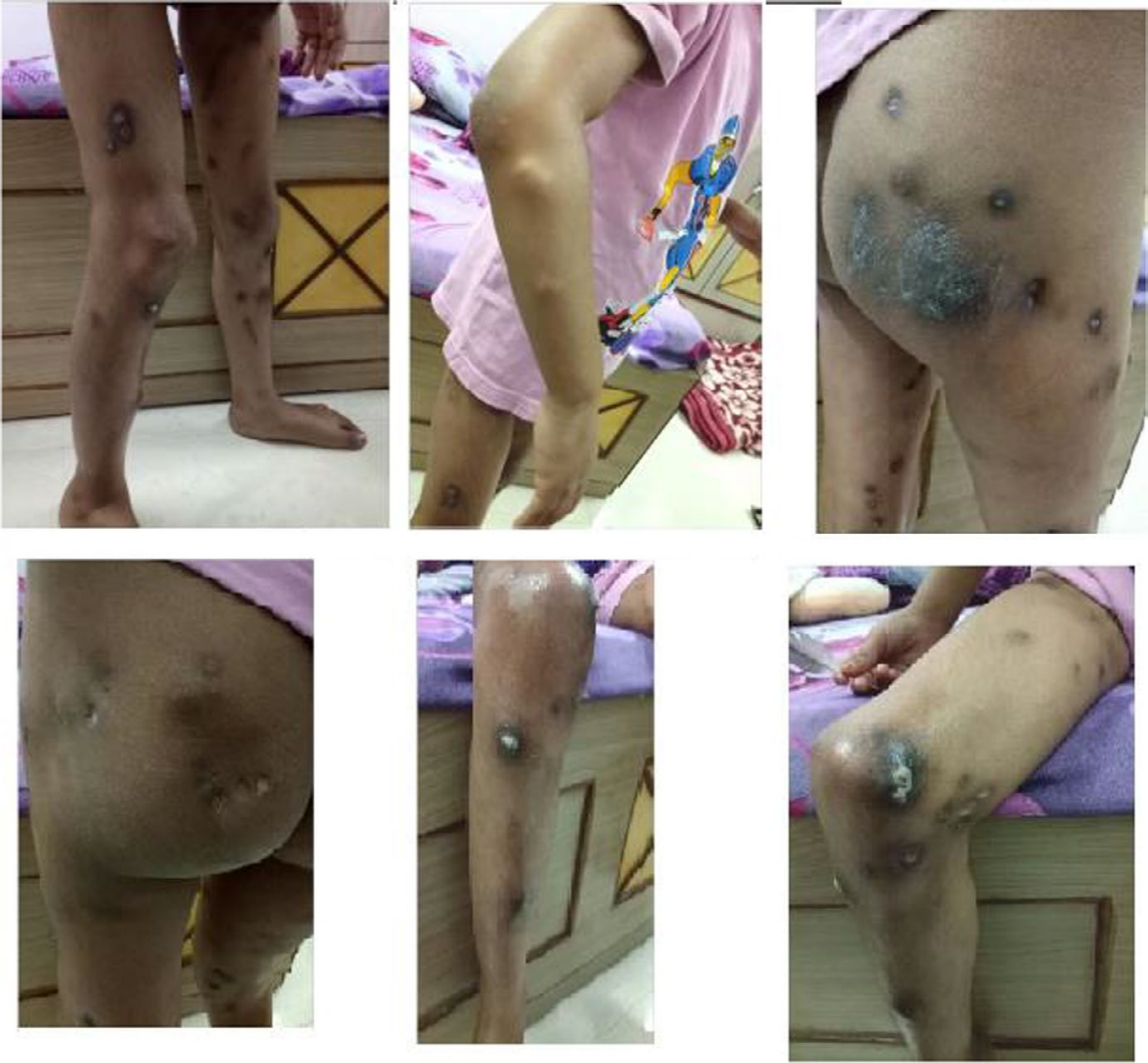
Clinical condition of the patient on first consultation

**Figure 3:**
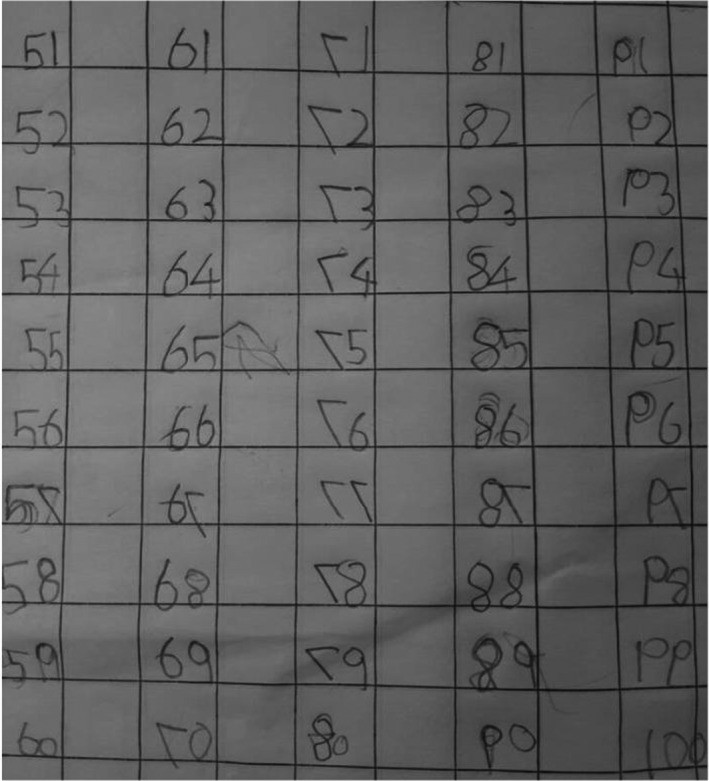
Writing pattern of the patient

On clinical examination, the patient suffered from obvious hard bony nodules on the face and both upper and lower limbs, which initially appeared at the age of 2.5 years. Those nodules were painful, especially during the night, with one or two new nodules appearing every month; the nodules discharged from time to time, leaving deep scars. Because of the nodules, the patient could not extend her right elbow and could not squat (Figure 2). She was a timid and reserved girl, with no relevant family history and her personal medical history was clear from any other major disease. Very interestingly when writing, she would make habitual mistakes, writing mirror images of the letters and numbers (Figure 3).

### 2.1 Treatment/Outcome

**Figure 4:**
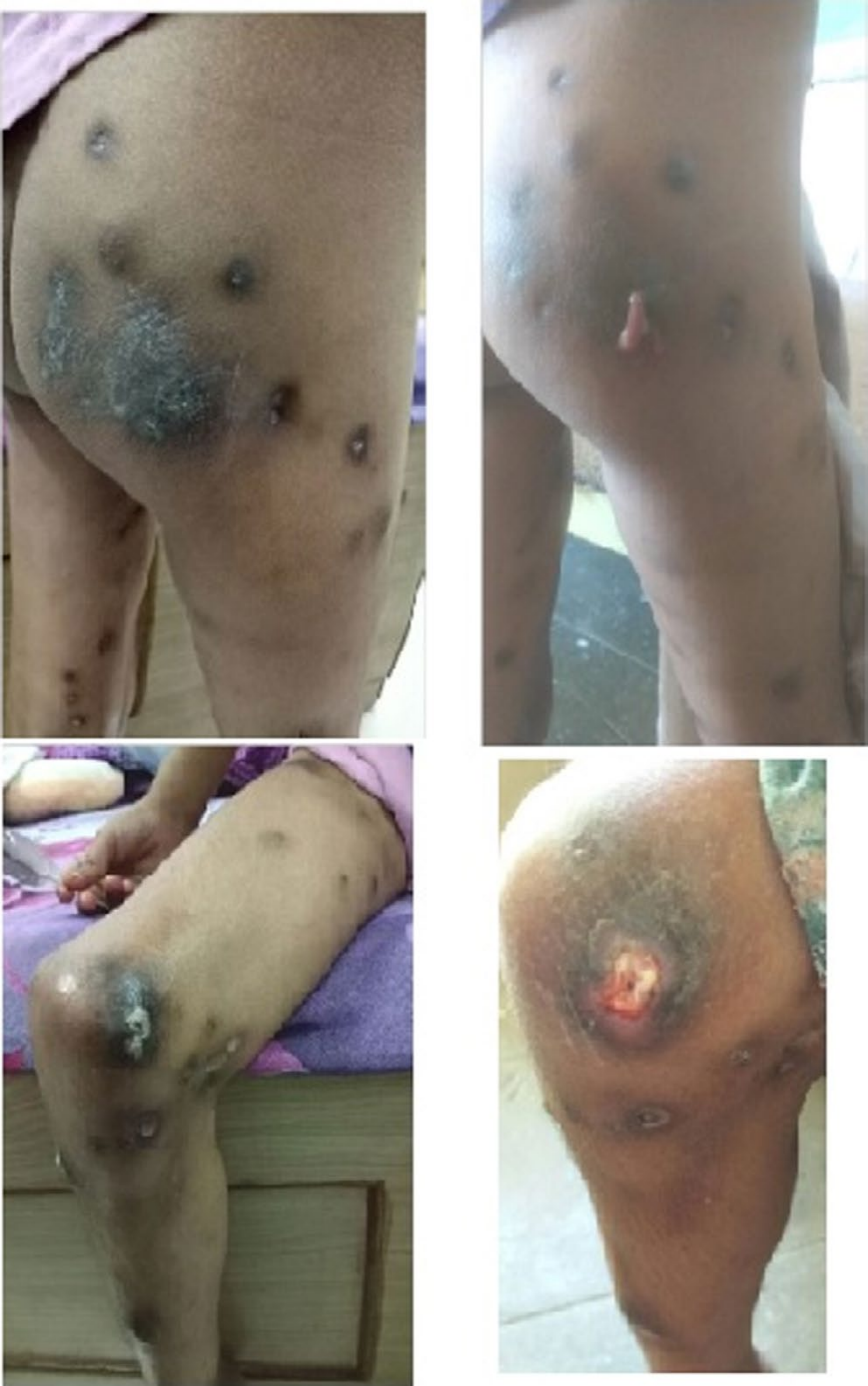
Discharging node of the right hip and knee 1 wk after first remedy

**Figure 5:**
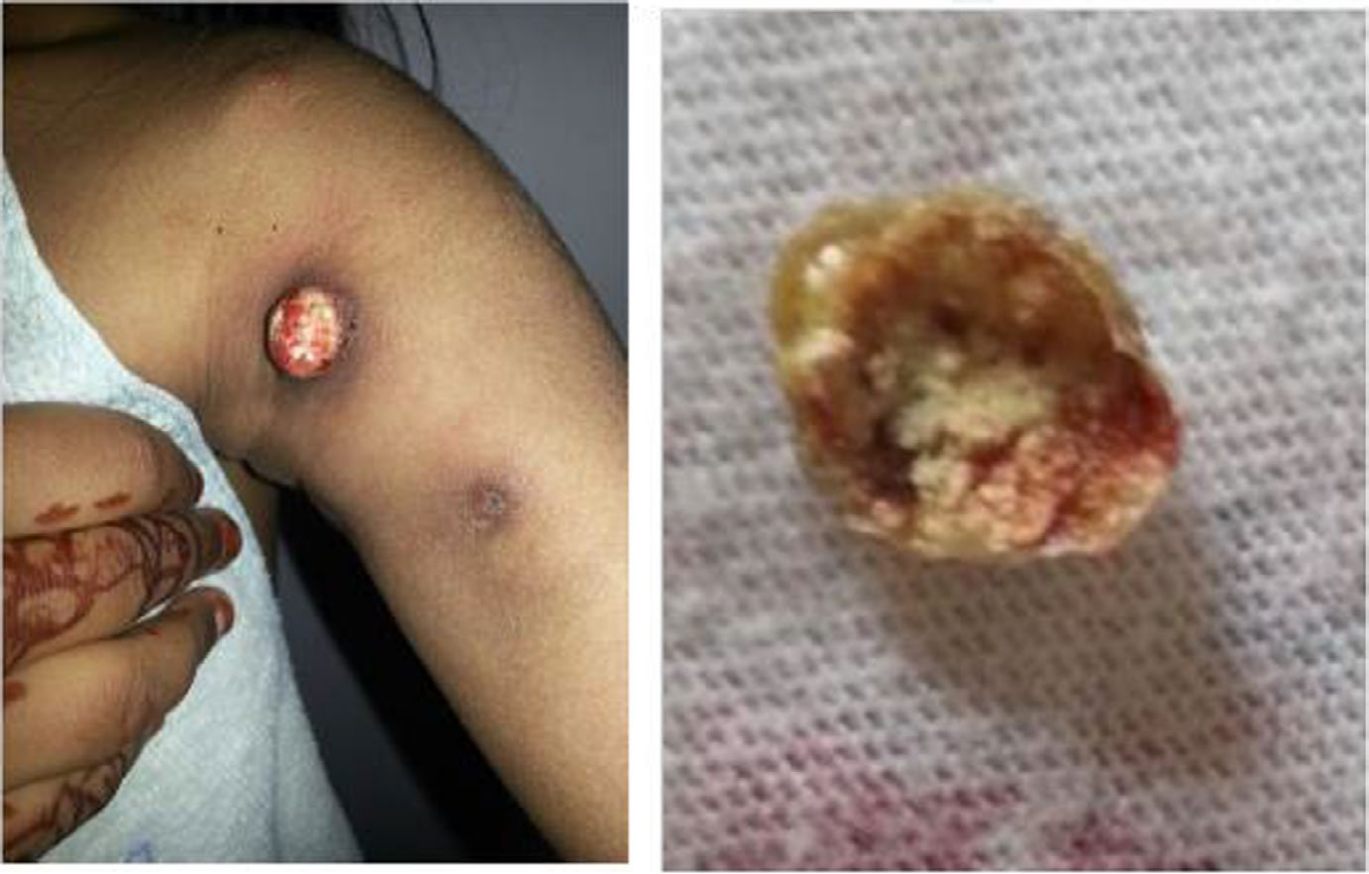
Calcific deposit expelled from left arm, 7 mo after initial treatment

The patient was treated with individualized remedies according to the principles of classical homeopathy and attended regular follow‐up visits. Table 1 presents the symptoms and the homeopathic remedies given to the patients over the course of her treatment. After the first month, her clinical picture was definitely improved: There were no more night pains, and the child was more active. There was no occurrence of any new nodes, and two old nodes became soft and discharged a lime‐colored discharge from the skin (Figure 4). Treatment with a series of remedies (Table 1), which were all prescribed according to the rules of classical homeopathy, continued over the following months, with slow but steady improvement of the condition of the patient. Seven months after the initial treatment, a calcific deposit was literally expelled from her left arm (Figure 5).

**Table 1:** Series of remedies prescribed to the patient during the course of her treatment

Date	Symptoms	Prescription
19 April 2017	Painful calcific nodes—symptoms worse during nighttime and from slight touch and motion. Offensive stools, urine, and breath odor. Reserved girl, making mistakes during writing, transposing letters. Desires salt and butter, dislikes sweets	Mercurius solubilis, 1M, one dose
20 May 2017	No night pains, gained 300 gr, more active. No new nodes. Two old nodes became soft and discharged lime‐like discharge	No remedy prescribed
21 August 2017	Desires sweet, weeping—tearful mood, timid when appearing in public. Offensive smell of stool with constipation. Dictatorial attitude. Still makes mistakes when writing, transposing letters	Lycopodium, 1M, one dose
10 February 2018	Patient continues to make mistakes in writing, lack of confidence, does not want to go to school	Repeat lycopodium 1M, one dose
23 September 2018	Nodules softened, as if ready to ooze—two of them red and very painful from slightest touch. Child cries because of pain. Hot head with cold extremities	Belladonna, 1M, one dose
19 December 2018	Offensive body discharges (stool, urine, perspiration), itching of the nodules, eats nasal crusts, disordered, desires sweets	Sulfur, 1M, one dose

**Figure 6:**
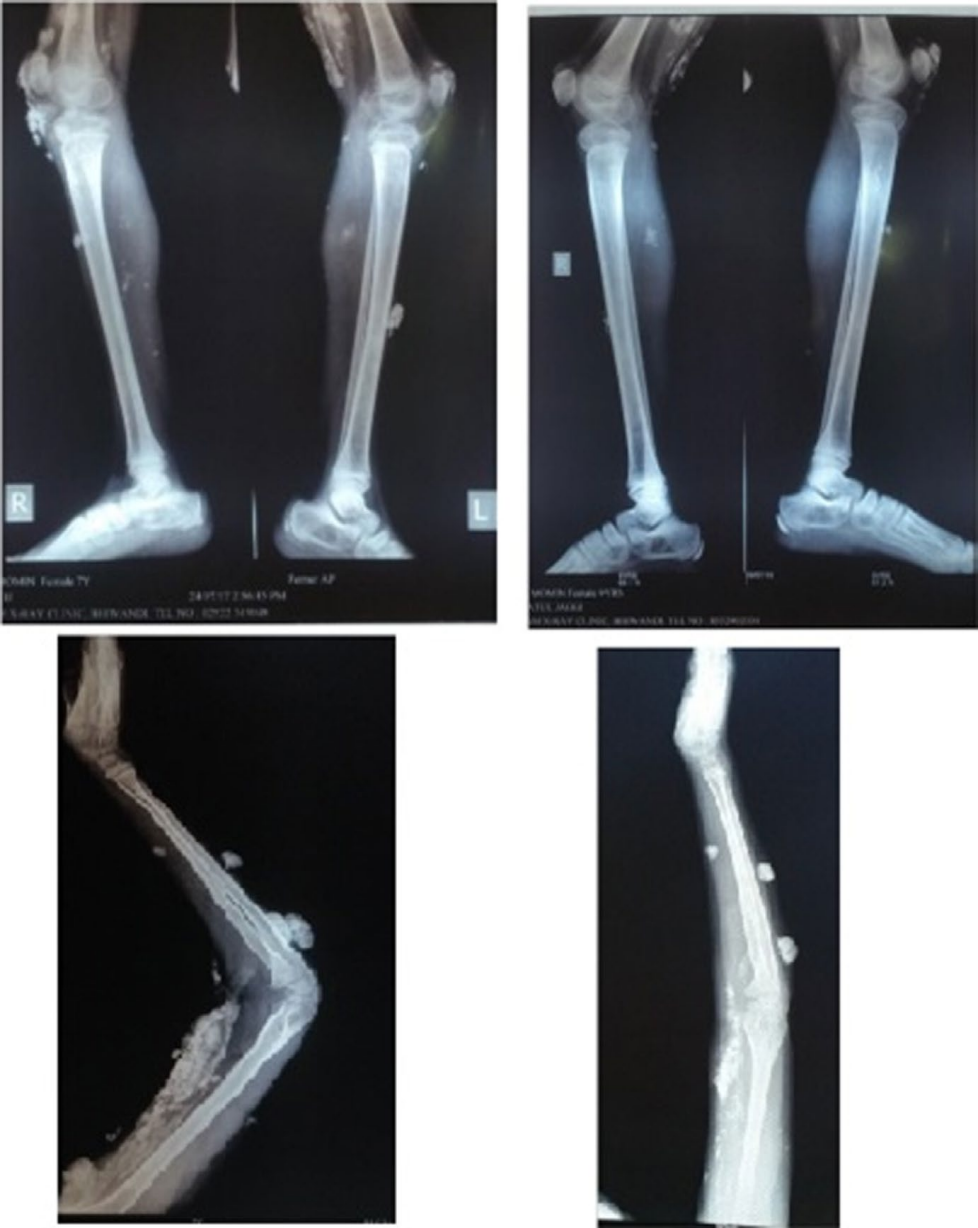
X‐rays of both legs and from right arm in the beginning of treatment and 15 mo later

**Figure 7:**
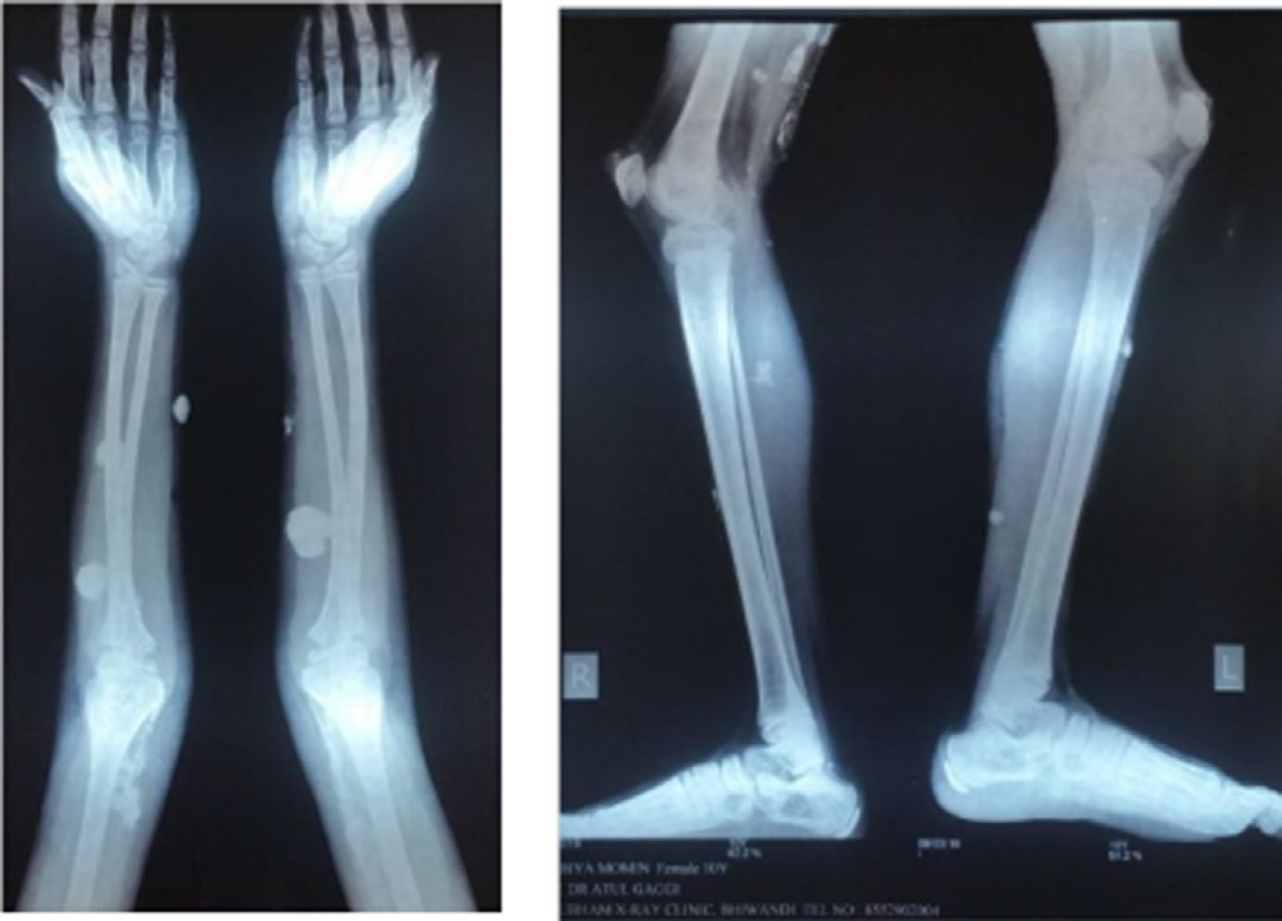
X‐rays, 23 mo after initial treatment

The radiological investigation 15 months after the initial treatment showed a significant reduction in the number and size of the calcific opacities (Figure 6). The patient could now squat and extend her right elbow joint. The radiological improvement was evident until the follow‐up visit and 23 months after the initial visit (Figure 7). The clinical condition of the patient was improving, and the laboratory studies were better: LDH 294.9 U/L (normal: 135‐214 U/L). The patient has not developed any new nodes since the beginning of the homeopathic treatment; actually we did not observe any new node after the first remedy. Additionally, we noticed improvement of her writing pattern; in the last follow‐up visit, the patient was not making any mistakes in writing at all.

**Figure 8:**
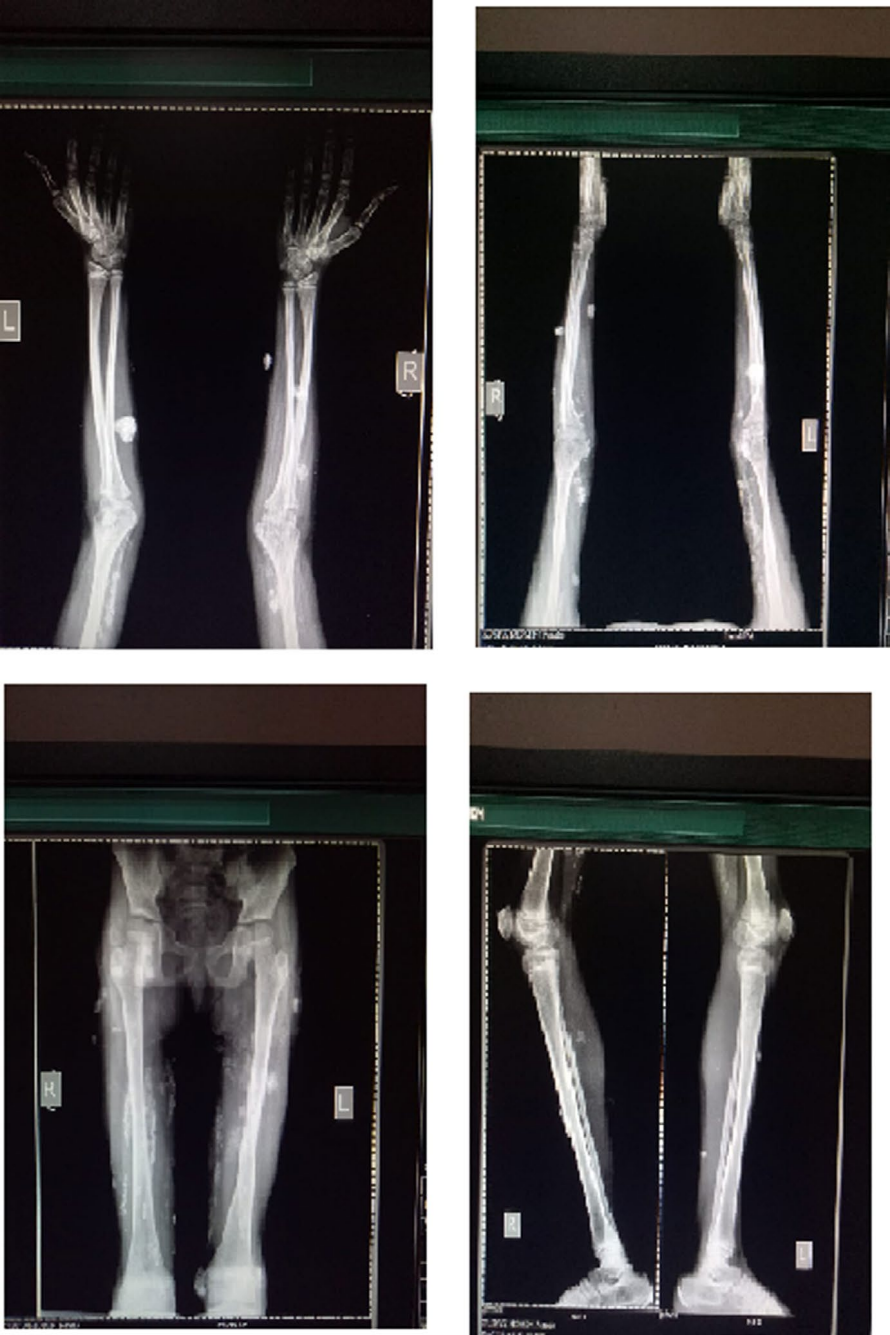
The latest X‐rays, 29 mo after initial treatment

The latest X‐rays of the patient were taken 29 months after the initial treatment (Figure 8). According to the Radiologist's report, “There is significant regression of soft tissue calcification in limbs as compared to previous films – No new lesion has appeared.”

## 3 DISCUSSION

Homeopathy, the “energy medicine,” founded in the nineteenth century by Dr Samuel Hahnemann (1755‐1843), is a branch of medical science based on the principle that diseases can be cured by strengthening the body's defense mechanism with substances selected for their energy‐giving properties. This observation is known as the *law of similar*s (Similia Similibus Curantur). Derived from the Greek words “homeo” and “pathos”, meaning “similar suffering,” homeopathy uses remedies, selected from herbs, minerals, or chemicals, which, in their crude form, would produce in a healthy body the same symptoms found in a sick person suffering from the specific disease. However, this crude substance is diluted and purified beyond the point of harm to its quintessential state of energy.^7^

The entire issue concerning health and disease hinges on the organism's ability to maintain homeostasis. The energy complex—also known as the “vital force,” according to Hahnemann^8^—is connected to the defense mechanism as a whole, being the foundation for optimal health and wellness, or lack thereof. When the energy complex is affected, imbalance of the organism is created. In time, this disparity has a great impact on the physical organism, causing disease.^9^

The extreme dilutions used in homeopathic remedies—dilutions beyond Avogadro's limit (<10^23^) and the assumption that substances diluted to that degree cannot contain active ingredient—have been the subject of heated controversy since Hahnemann's time, leading to the argument that homeopathic remedies contain no active ingredient and are, therefore, inert. Nevertheless, recent research proves the opposite. Chickramane et al (2010) were the first to discover the presence of nanoparticle source materials of metal‐derived homeopathic medicines in multiple ultradilutions beyond Avogadro's number by using transmission electron microscopy (TEM), electron diffraction, and chemical analysis by inductively coupled plasma‐atomic emission spectroscopy (ICP‐IES).^10^ Even more recently, Tournier and Roberts in 2015 experimented with solvatochromic dyes and showed that the presence of homeopathic dilution glycerol 50M consistently and reproducibly affected the absorption spectra of all six solvatochromic dyes tested when compared to the control, meaning that homeopathic dilutions are not “just water” 11, 12, 13 !

The pathogenesis of acquired HO is not clear, but it is thought that severe local inflammation triggered by the physical insult leads to the recruitment of progenitor cells, the release of proskeletogenic factors, the derangement of the normal tissue repair processes, and, finally, the formation of heterotopic bone.^1^ However, congenital HO is very rare, but can be very severe, occurring in children with fibrodysplasia ossificans progressiva (FOP) or progressive osseous heteroplasia (POH). Several mutations in these pathological conditions have been identified, and they all cluster in the intracellular glycine‐serine (GS)‐rich domain of ALK2, the most common of these mutations being the ACVR1^R206H^ 14.

Congenital HO could be truly difficult to treat. In FOP patients, the pathology can be very aggressive, involving the accumulation of large amounts of endochondral bone masses throughout the body. HO in those patients is inoperable, since the disease is highly reactive and surgery could cause recurrent and even more severe HO. During the exacerbations of the symptoms, high doses of corticosteroids for a brief period of 4‐5 days are indicated. Steroid treatment can alleviate inflammation, swelling, and pain, but cannot reduce the progression of HO.^15^ Since FOP patients carry mildly activating mutations, therapeutic strategies, over the last few years, have been experimenting with various drugs directed toward specific paths in the pathogenic cascade of the disease.^1^

Both the clinical and the radiological results of our patient after her treatment with individualized classical homeopathy are definitely promising. According to Vithoulkas, the defense mechanism as a whole appears to have a “higher” intelligence that is able to maintain optimum balance under any stress. Homeopathy can treat diseases with milder means, which promote and enhance the natural reaction of the immune system.^16^, ^17^, ^18^

## 4 CONCLUSION

We present a case of a young girl suffering from extensive congenital heterotopic ossification whose treatment according to the principles of classical homeopathy had very good clinical and radiological results. This is the first published case concerning the treatment of this difficult pathology with classical homeopathy. Many more cases and much more research are definitely needed in order to conclude that classical homeopathy can be a treatment option for this severe pathology.

## CONFLICT OF INTEREST

The authors have no conflict of interest to declare.

## AUTHOR CONTRIBUTIONS

Dr JA and Dr LJ: were responsible for the treatment of the patient, under the supervision of Prof. GV. Dr SM and Dr DT: underwent the literature review and the writing of the paper.

## References

[1] Pacifici M . Acquired and congenital forms of heterotopic ossification: new pathogenic insights and therapeutic opportunities. Curr Opin Pharmacol. 2018;40:51‐58. 29614433 10.1016/j.coph.2018.03.007 PMC6015534

[2] van Kuijk AA , Geurts AC , van Kuppevelt HJ . Neurogenic heterotopic ossification in spinal cord injury. Spinal Cord. 2002;40:313‐326. 12080459 10.1038/sj.sc.3101309

[3] Bossche LV , Vanderstraeten G . Heterotopic ossification: a review. J Rehabil Med. 2005;37:129‐136. 16040468 10.1080/16501970510027628

[4] Jitariu A , Hersdea R , Ceausu A . Myositis ossificans‐a case report and review of literature. Research and Clinical Medicine. 2016;1:26‐29.

[5] Li PF , Lin ZL , Pang ZH . Non‐traumatic myositis ossificans circumscripta at elbow joint in a 9‐year old child. Chin J Traumatol. 2016;19:122‐124. 27140223 10.1016/j.cjtee.2016.01.009 PMC4897847

[6] Murrad K , Rand A , Abdulaziz J , Mrad MA . Heterotopic ossification in a newborn: a case report. Eplasty. 2016;16:e37. 28344729 PMC5360091

[7] Vithoulkas G . The science of homeopathy. Athens, Greece: International Academy of Classical Homeopathy; 2012.

[8] Hahnemann S . Organon der rationellen Heilkunst. Leipzig (Arnold), Germany: Stuttgart Homoion‐Verlag; 1810.

[9] George V . Levels of health. The second volume of the science of homeopathy. Alonissos, Greece: International Academy of Classical. Homeopathy 2019.

[10] Chikramane PS , Suresh AK , Bellare JR , Kane SG . Extreme homeopathic dilutions retain starting materials: a nanoparticulate perspective. Homeopathy. 2010;99:231‐242. 20970092 10.1016/j.homp.2010.05.006

[11] Tournier A , Roberts R . Chemical dyes can detect presence of homeopathic high dilutions. London, UK: HRI Research Article; 2015.

[12] Mahesh S , Shah V , Mallappa M , Vithoulkas G . Psoriasis cases of same diagnosis but different phenotypes‐management through individualized homeopathic therapy. Clin Case Rep. 2019;7:1499‐1507. 31428376 10.1002/ccr3.2197 PMC6693058

[13] Mahesh S , Mallappa M , Vithoulkas G . Gangrene: five case studies of gangrene, preventing amputation through homoeopathic therapy. Ind J Res Homoeopathy. 2015;9:114‐122.

[14] Pacifici M , Shore EM . Common mutations in ALK2/ACVR1, a multi‐faceted receptor, have roles in distinct pediatric musculoskeletal and neural orphan disorders. Cytokine Growth Factor Rev. 2016;27:93‐104. 26776312 10.1016/j.cytogfr.2015.12.007 PMC4753137

[15] Kaplan FS , Shore EM , Glaser DL , Emerson S . The medical management of fibrodysplasia ossificans progressiva: current treatment considerations. Clin Proc Int Clin Consort Fibrodysplasia Ossificans Progressiva. 2011;4:1‐100.

[16] Vithoulkas G , Carlino S . The "continuum" of a unified theory of diseases. Med Sci Monit. 2010;16:7‐15. 20110932

[17] Mahesh S , Mallappa M , Tsintzas D , Vithoulkas G . Homeopathic treatment of vitiligo: a report of fourteen cases. Am J Case Rep. 2017;18:1276‐1283. 29196612 10.12659/AJCR.905340 PMC5723025

[18] Chabanov D , Tsintzas D , Vithoulkas G . Levels of health theory with the example of a case of juvenile rheumatoid arthritis. J Evid Based Integr Med. 2018;23. 10.1177/2515690X18777995 PMC6024340 29896977

